# Supplementary material for: Temporal genome-wide fitness analysis of Mycobacterium marinum during infection reveals the genetic requirement for virulence and survival in amoebae and microglial cells
Source: mSystems. 2024 Jan 25;9(2):e01326-23. doi: 10.1128/msystems.01326-23 (PMC10878075; doi:10.1128/msystems.01326-23)
Supplement: Legends — Supplemental figure legends. [file msystems.01326-23-s0001.docx]

**Table S1 Insertion counts per TA site for *D. discoideum* and BV2 experiments (**<https://tinyurl.com/5dbxww5w>)

**Table S2 Gene counts for *D. discoideum* and BV2 experiments**

**Table S3 Conditional essentiality analysis for *D. discoideum* and BV2 experiments**

**Table S4 Enrichment analyses for *D. discoideum* and BV2 experiments**

**File S1**

**Supplementary Figures**

**Fig. S1. PCA of all experimental conditions in *D. discoideum* and library saturation**

**A**: Principal components 1 and 2 of normalized insertion counts, coloured by conditions: Inoculum (n=7), 24 hpi (n=6), 48 hpi (n=8), Inoculum Bis (n=6) and 2x48 hpi (n=6). A small difference is observed between 48 hpi and Inoculum_bis samples, which might be due to sample manipulation, mainly freezing, thawing and cultivation in 7H9 of the 48 hpi samples to prepare the Inoculum_bis. This difference was regarded as negligible, compared to the benefit of comparing all selection conditions to the same Inoculum condition. **B** depicts the density of the percentage of disrupted site per genes with a normalized count of at least 1. The peak of essential genes is well distinguishable from the rest of the genes and is comparable between experimental conditions.

**Fig. S2. Differential representation of *M. marinum* mutants**

KEGG terms enriched in the essential core plotted in an enrichment map. Using R and the package clusterProfiler terms were filtered by *p*-value < 0.05. The *p*-value is colour-coded, and the number of enriched genes found in the respective term are coded by dot size. Edges between nodes represent overlap between the connected terms.

**Fig. S3 Hierarchical clustering of log_2_ fold changes in *D. discoideum***

**A,** **B, C**. Enriched GO terms in cluster 2, 7 and 9 depicted as dot plot. Using R and the package clusterProfiler terms were filtered by *p*-value < 0.01. The *p*-value is colour-coded, and the number of enriched genes found in the respective term are coded by dot size. GeneRatio equals the number of differentially expressed genes against the number of genes associated with a GO term in *M. marinum* genome.

**Fig. S4. Fitness advantage (FA) and fitness disadvantage (FD) over the infection time course in *D. discoideum***

Genes thresholded as described in the results part, were submitted to GO term enrichment using R and the package clusterProfiler. Terms were filtered by *p*-value < 0.01 and visualized as dotplots. The *p*-value is colour-coded and the number of enriched genes per term is coded by the dot size. **A.** Enriched GO terms for FA and FD at 24 hpi **B.** Enriched GO terms FA and FD at 48 hpi **C.** Enriched GO terms FA at 2x48 hpi **D.** Enriched GO terms FD at 2x48 hpi. GeneRatio equals the number of differentially expressed genes against the number of genes associated with a GO term in *M. marinum* genome. **E, F, G.** Volcano plots of binary comparisons of 24 hpi, 48 hpi and 2x48 hpi respectively to the Inoculum. The x-axis shows the log_2_fc and the y-axis the negative decadic logarithm of the associated *p*-values. The lowest thresholds used to filter genes for enrichment analysis are depicted as thick black lines: log_2_fc ≥ 0.585 and ≤ 0.585 (corresponding to 1.5-fold change) and *p*-value ≤ 0.1. For visualization *p*-values < 0.0001 were capped to 0.0001.

**Fig. S5. Clustering of differentially affected genes of *M. marinum* during infection of BV2 microglial cells**

Plots of principal component analysis of the *M. marinum* Inoculum (n=4) versus 48 hpi (n=3) of BV2 infection.
