# Supplementary figures and images for: Temporal genome-wide fitness analysis of Mycobacterium marinum during infection reveals the genetic requirement for virulence and survival in amoebae and microglial cells
Source: mSystems. 2024 Jan 25;9(2):e01326-23. doi: 10.1128/msystems.01326-23 (PMC10878075; doi:10.1128/msystems.01326-23)

A

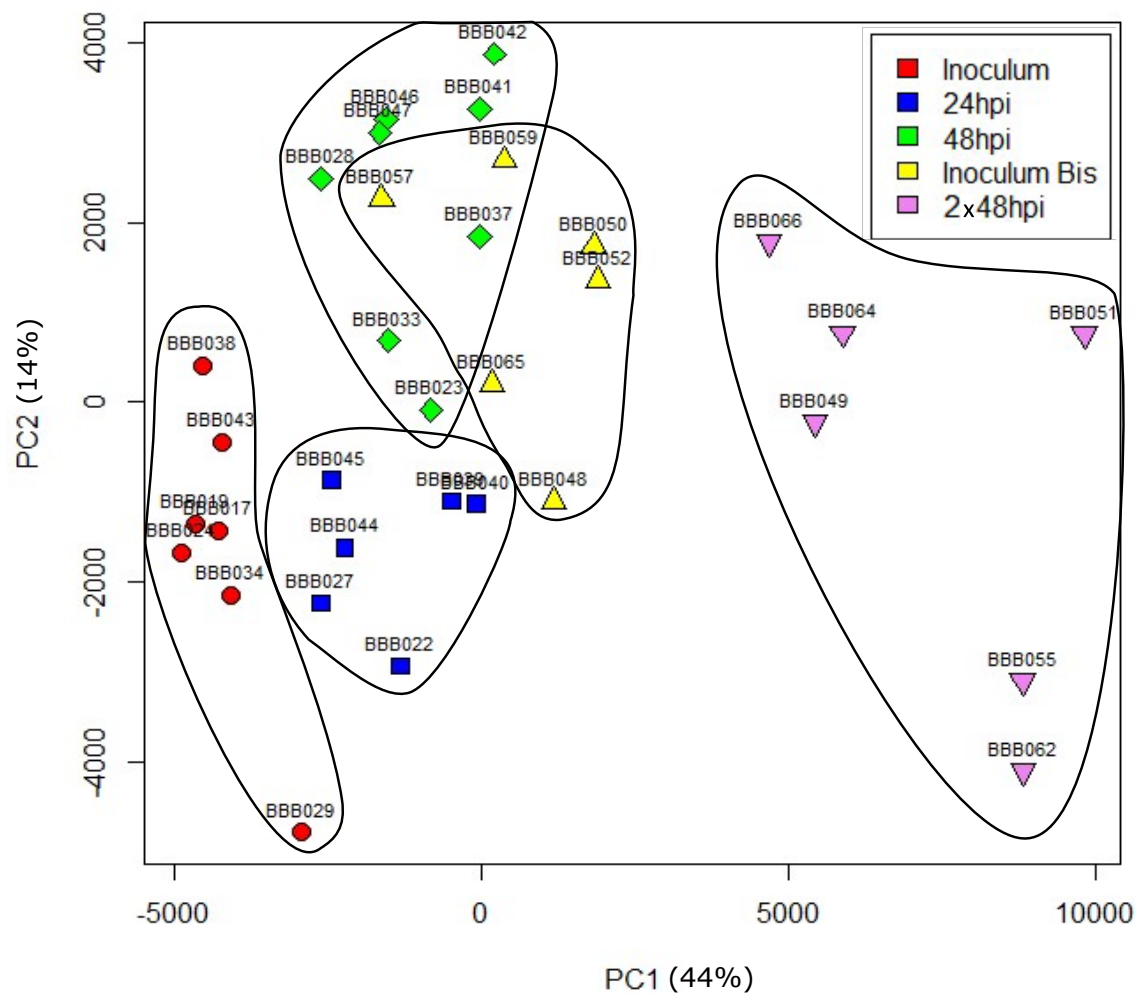

B

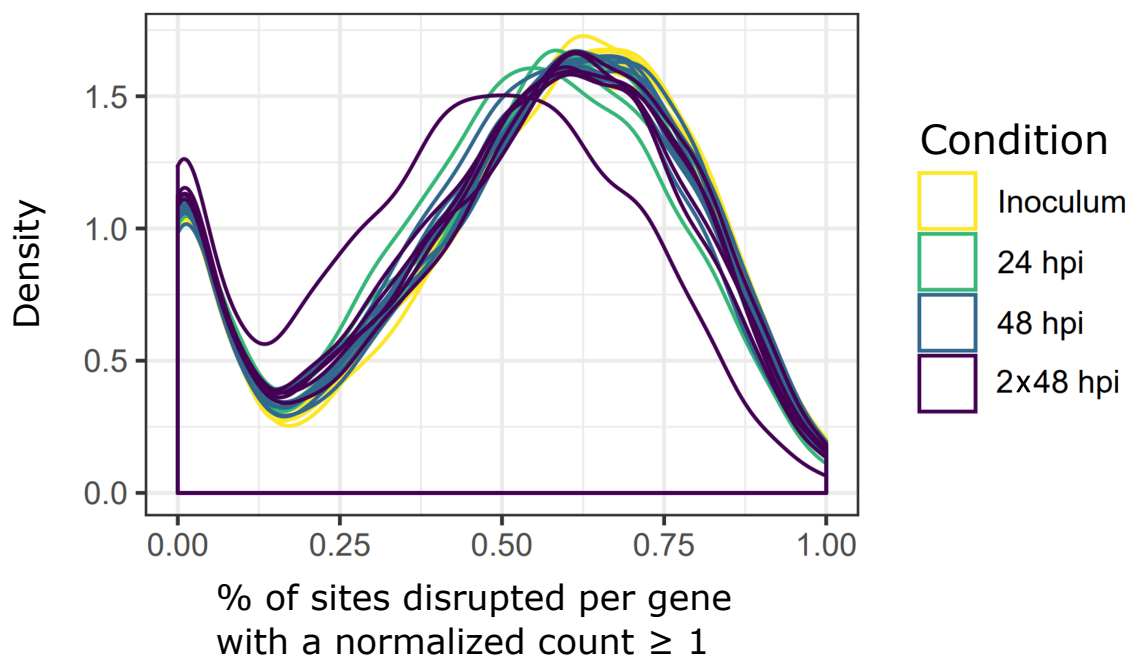

Supplement: Figure S1 — PCA of all experimental conditions in D. discoideum and library saturation. [file msystems.01326-23-s0002.pdf]

A

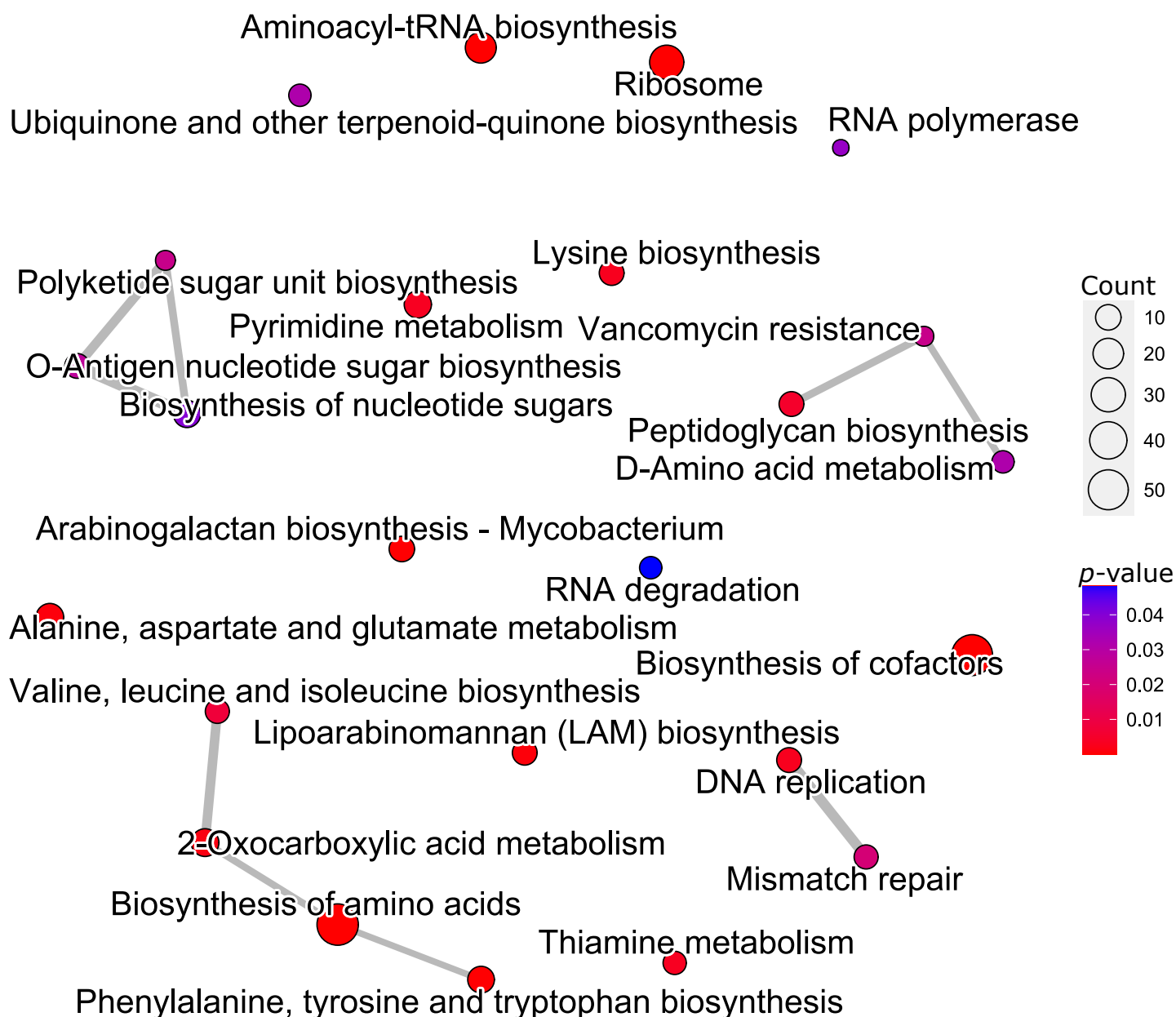

Supplement: Figure S2 — Differential representation of M. marinum mutants [file msystems.01326-23-s0003.pdf]

## A Cluster 2

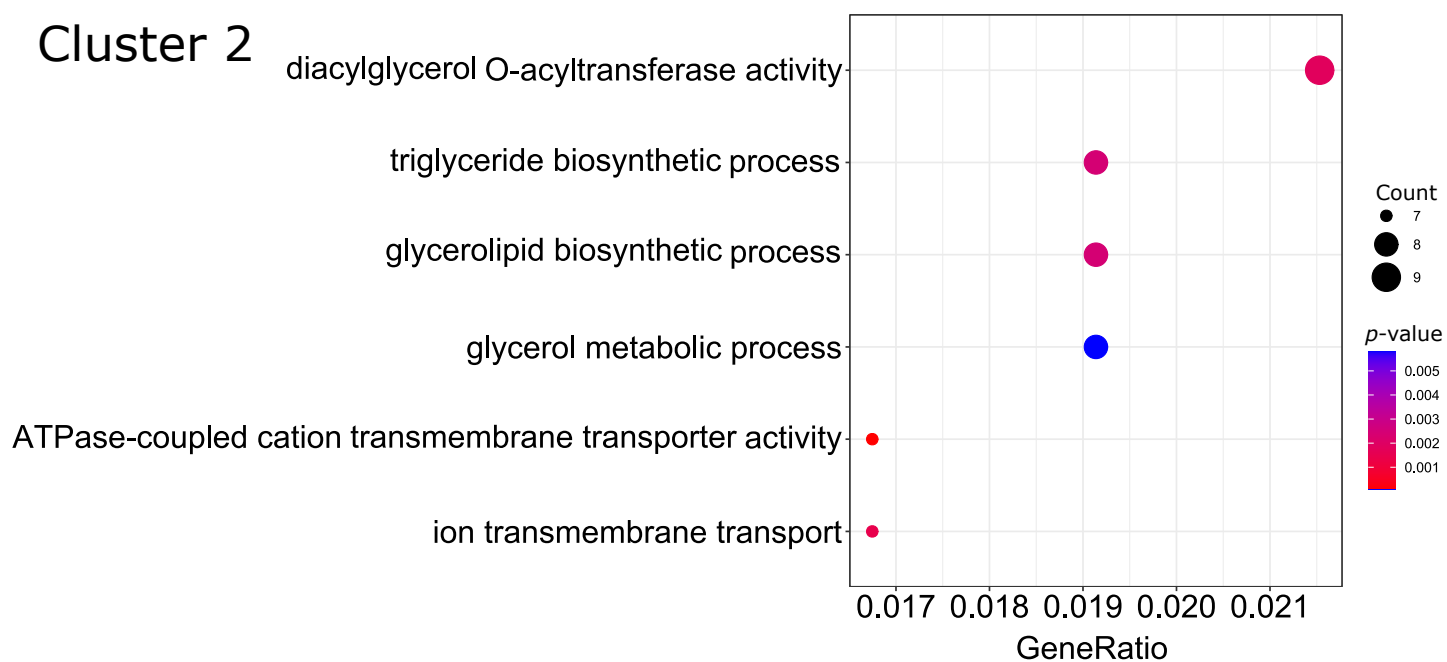

## B Cluster 7

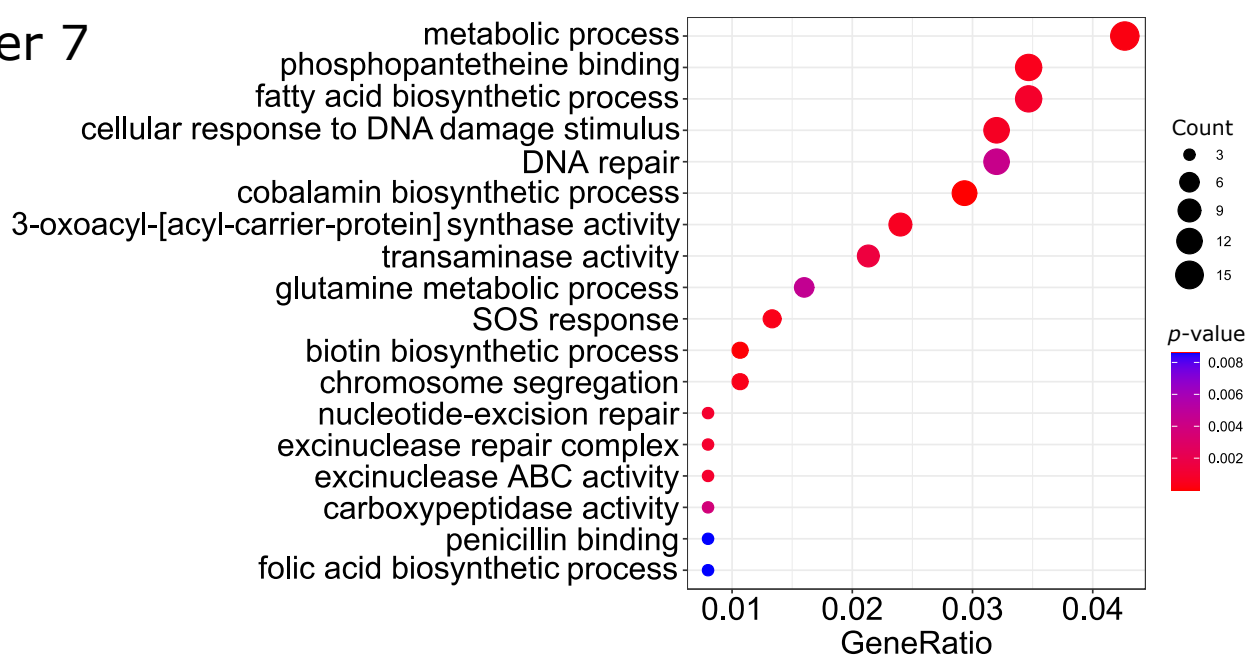

## C Cluster 9

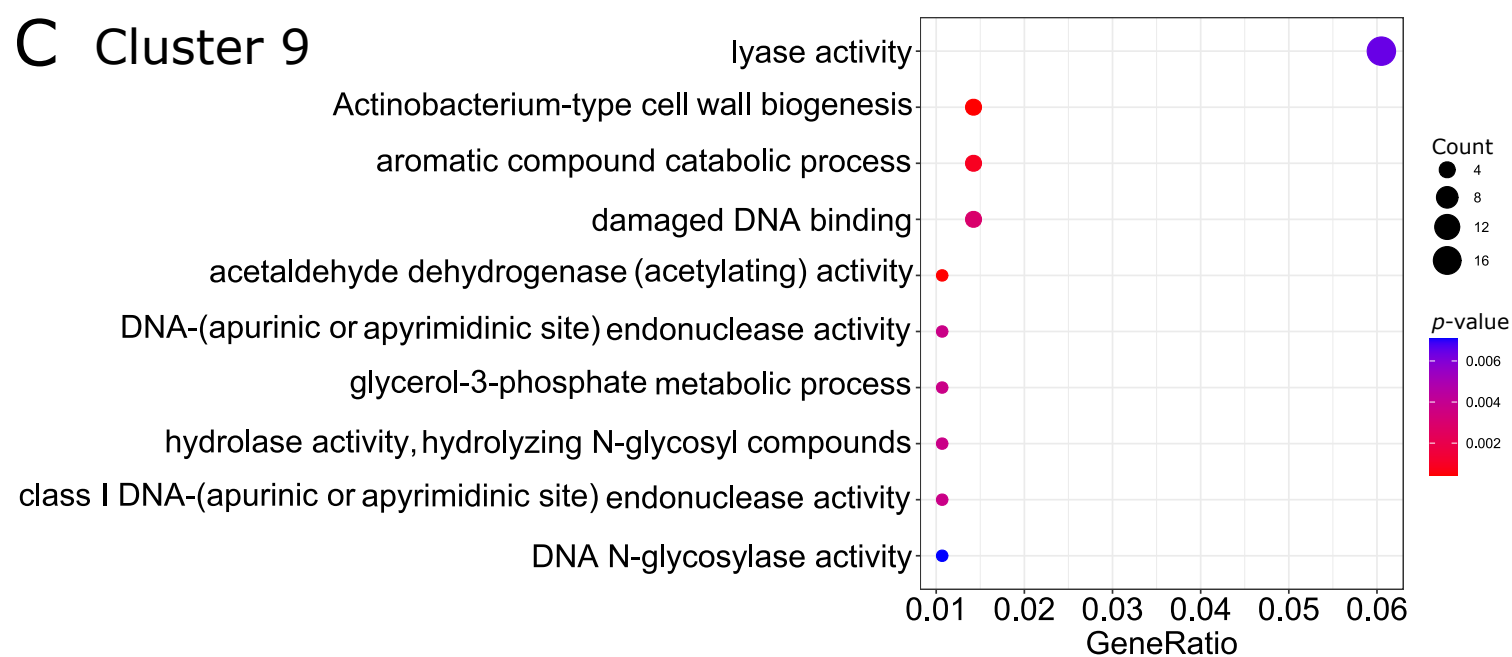

Supplement: Figure S3 — Hierarchical clustering of log2 fold changes in D. discoideum. [file msystems.01326-23-s0004.pdf]

A

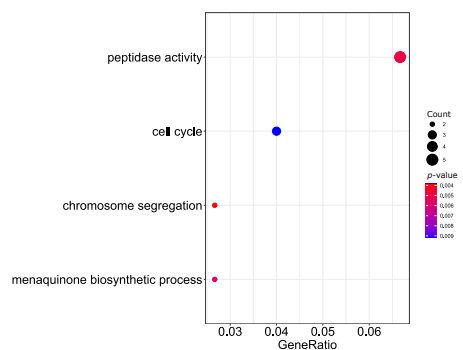

B

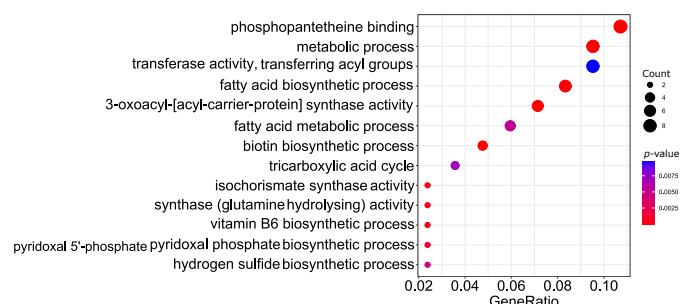

C

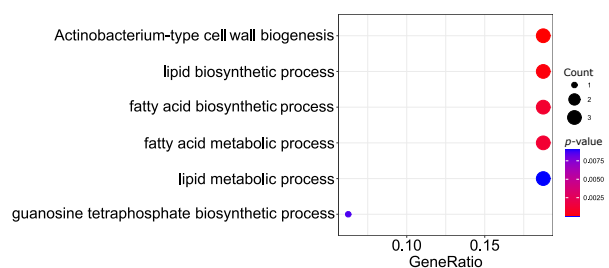

E

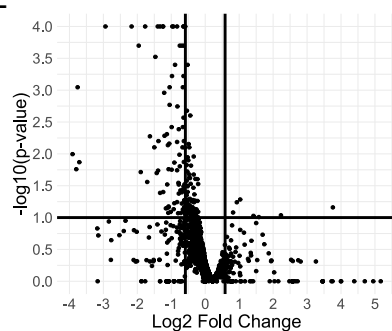

F

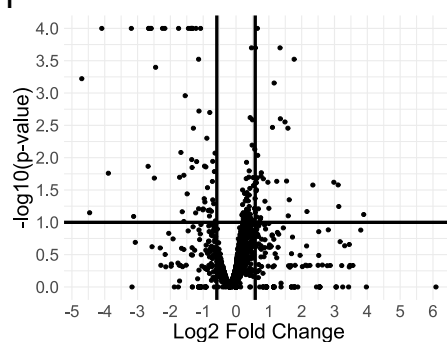

G

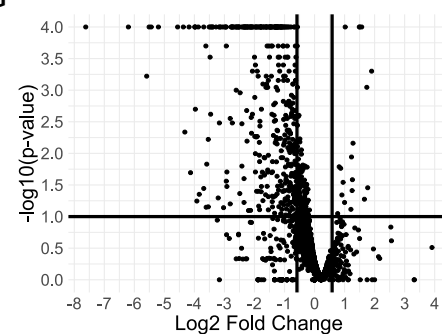

D

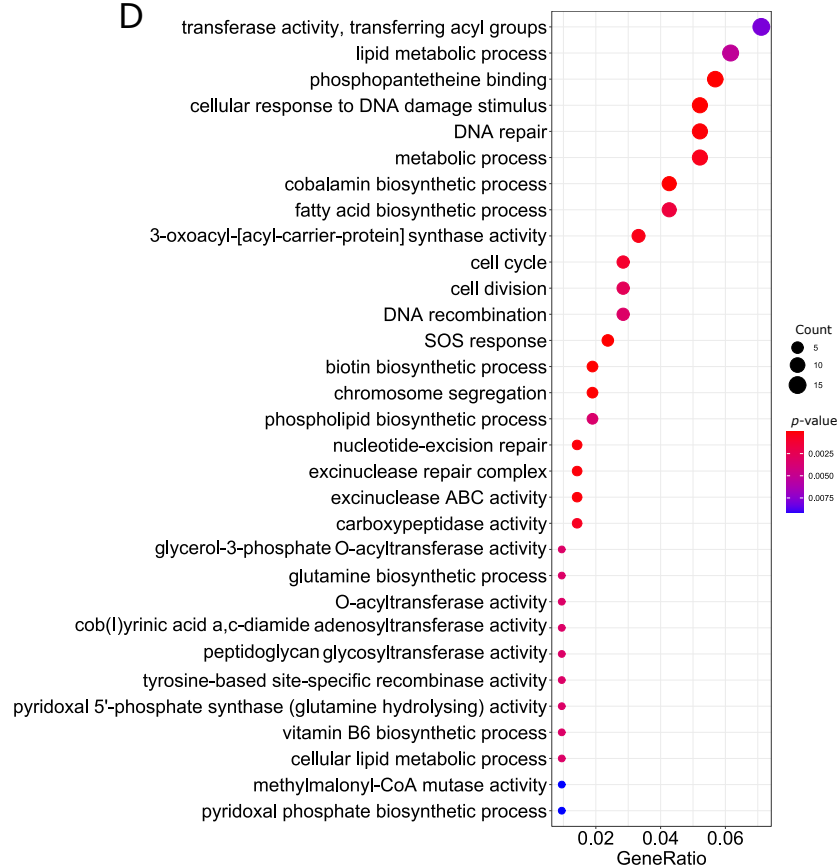

Supplement: Figure S4 — Fitness advantage and fitness disadvantage over the infection time course in D. discoideum. [file msystems.01326-23-s0005.pdf]

Lefrançois et al., Figure S5

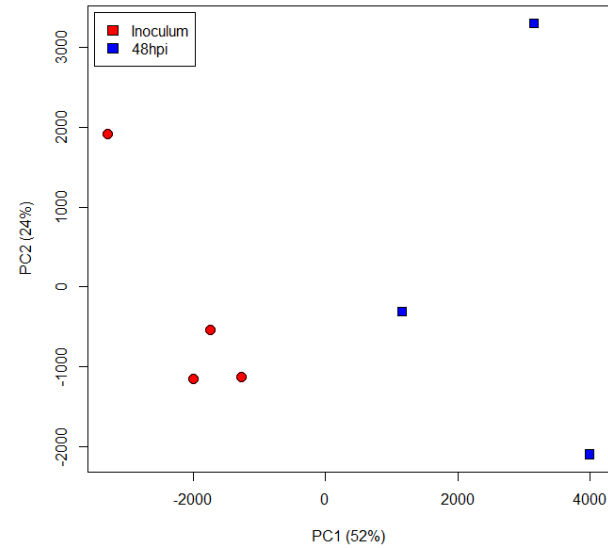

Supplement: Figure S5 — Clustering of differentially affected genes of M. marinum during infection of BV2 microglial cells. [file msystems.01326-23-s0006.pdf]
